# *MMiRNA-Viewer*^*2*^, a bioinformatics tool for visualizing functional annotation for MiRNA and MRNA pairs in a network

**DOI:** 10.1186/s12859-020-3436-7

**Published:** 2020-07-06

**Authors:** Yongsheng Bai, Steve Baker, Kevin Exoo, Xingqin Dai, Lizhong Ding, Naureen Aslam Khattak, Hongtao Li, Hannah Liu, Xiaoming Liu

**Affiliations:** 1 Department of Biology, Indiana State University, Terre Haute, IN 47809 USA; 2 Department of Internal Medicine, University of Michigan, Ann Arbor, MI 48109 USA; 3 Department of Mathematics and Computer Science, Indiana State University, Terre Haute, IN 47809 USA; 4 Department of Science and Mathematics, St Mary-of-the-Woods College, Saint Mary of the Woods, IN 47876 USA; 5 Carmel High School, 520 E. Main St., Carmel, IN 46032 USA; 6 USF Genomics, College of Public Health, University of South Florida, Tampa, FL 33620 USA

**Keywords:** miRNA, mRNA, Gene regulation, Clustering algorithm, The cancer genome atlas, Target prediction, Visualization, *MMiRNA-Viewer*^*2*^

## Abstract

**Background:**

Although there are many studies on the characteristics of miRNA-mRNA interactions using miRNA and mRNA sequencing data, the complexity of the change of the correlation coefficients and expression values of the miRNA-mRNA pairs between tumor and normal samples is still not resolved, and this hinders the potential clinical applications. There is an urgent need to develop innovative methodologies and tools that can characterize and visualize functional consequences of cancer risk gene and miRNA pairs while analyzing the tumor and normal samples simultaneously.

**Results:**

We developed an innovative bioinformatics tool for visualizing functional annotation of miRNA-mRNA pairs in a network, known as *MMiRNA-Viewer*^*2*^. The tool takes mRNA and miRNA interaction pairs and visualizes mRNA and miRNA regulation network. Moreover, our *MMiRNA-Viewer*^*2*^ web server integrates and displays the mRNA and miRNA gene annotation information, signaling cascade pathways and direct cancer association between miRNAs and mRNAs. Functional annotation and gene regulatory information can be directly retrieved from our web server, which can help users quickly identify significant interaction sub-network and report possible disease or cancer association. The tool can identify pivotal miRNAs or mRNAs that contribute to the complexity of cancer, while engaging modern next-generation sequencing technology to analyze the tumor and normal samples concurrently. We compared our tools with other visualization tools.

**Conclusion:**

Our *MMiRNA-Viewer*^*2*^ serves as a multitasking platform in which users can identify significant interaction clusters and retrieve functional and cancer-associated information for miRNA-mRNA pairs between tumor and normal samples. Our tool is applicable across a range of diseases and cancers and has advantages over existing tools.

## Background

Among potential new diagnostic and therapeutic targets, microRNAs (miRNAs) have been increasingly studied because they play a well-conserved and crucial role in normal biological processes, such as cellular differentiation, proliferation, and apoptosis through a complex gene regulation networking. miRNAs are involved in post-transcriptional regulation of the target mRNA through binding to the 3′ UTRs of messenger RNAs (mRNAs) and playing roles of degradation of target mRNA or suppression of protein translation [1]. Thus, the co-regulation of miRNA affects the function of mRNAs in multiple ways or vice versa [2]. The understanding towards these mechanisms significantly increases with the advent of high-throughput expression profiling. In this context, the correlation of miRNA-mRNA could represent a meaningful biological signal that might be annotated systematically with automated data mining techniques [3].

In recent years, many tools have been developed to explore and visualize the miRNA and mRNA interactions to gain a deep insight of the disease’s association in terms of functional characteristics of the related miRNA-mRNA interactions. For example, a set of genes that show strong connections with miRNA regulation should reveal the critical signaling pathways that are activated or deactivated during the target miRNA-mRNA interplay. Some tools are discussed as follows.

Utilizing expression correlation or up/down regulation of miRNA and mRNA from their expression data and utilizing miRNA-mRNA targeting databases or prediction algorithms, some tools like MiRontop [4], DIANA-mirExTra [5], MAGIA [6], CoMeTa [7], and mirConnX [8] can build regulatory network of miRNA, mRNA, and transcription factors. It is noteworthy that another miRNA-mRNA regulatory network construction tool, miRGator [9], utilizes publicly available deep sequencing miRNA data to increase the accuracy of miRNA–targeting relationships. The built regulatory network is usually visualized in graph, followed by functional analysis of genes and/or miRNAs wherein. The functional analysis usually refers to GO enrichment, KEGG pathway, function association to diseases, etc. DIANA-miRPath [10] is such a tool for functional analysis. The available tools for GO term enrichment include GoMiner [11], FatiGO [12], BiNGO [13], GOAT [14], DAVID [15], CytoScape plug-in BiNGO [13], GOLEM [16], GOEAST [17], and GOTM [18], GSEA [19], FatiScan [20], GO-stat [21], GeneTrail [22], and iGA [23].

However, these foregoing tools, namely MiRontop [4], DIANA-mirExTra [5], MAGIA [6], CoMeTa [7], and mirConnX [8], and miRGator [9], cannot meet the research need of characterizing the change of miRNA and mRNA regulatory networks from normal to tumor in the Cancer Genome Atlas (TCGA) studies for reasons described below. First, these tools can only build miRNA-mRNA regulatory network in one condition, eg., a specific tumor. Thereby, each edge in the regulatory network has only one weight accordingly. Second, the graphs generated by tools like MAGIA2 [24] are static and non-interactive. Thereby, it’s inconvenient for users to manipulate the graph to retrieve useful information. Third, these tools only construct the miRNA-mRNA regulatory network by rendering the connected miRNA and mRNAs that are filtered based on differential expression, expression coefficient, or targeting site analysis. Thereby, these tools lack a underlying algorithm that can further cluster the miRNAs and mRNAs to reveal the complexity of the miRNA and mRNA interaction [25].

Not only integrating expression data and targeting information of miRNA and mRNA, some tools also provide the clustering algorithm to find common causal factors in the complex direct and indirect miRNA-mRNA regulatory network. For example, miRMAP [26] applies bicluster analysis to reveal the miRNA-mRNA functional modules using integrated data set and visualizes a network of miRNA-mRNA modules that are enriched for both miRNA classes and biological processes. miRMAP investigates both significant positive and negative correlation coefficients between miRNAs and mRNAs. However, miRMAP only considers one condition, for example, tumor condition. Thereby it cannot consider and visualize the correlation coefficient alteration of miRNA and mRNA from normal to tumor.

To overcome the shortcomings of the above-mentioned tools, in our previous studies, a dynamic visualization tool, *MMiRNA-Viewer* (http://bioinf1.indstate.edu/mmirna-viewer/), was developed to explore the miRNA and mRNA TCGA expression data. The graph produced by *MMiRNA-Viewer* shows two kinds of edges/links between each pair of miRNA-mRNA nodes to represent simultaneously the expression correlation coefficients between mRNA-miRNA pairs in two conditions, namely in normal and in tumor samples. Users have several custom filters like miRNA-mRNA targeting relationship validation databases, expression fold change and so on, so that interesting subgraphs of the miRNA and mRNA regulatory network can be highlighted. Furthermore, Louvain algorithm [27] is provided as an underlying clustering algorithm to let users to find miRNA and mRNA clusters.

In this study, we presented a more comprehensive and user-friendly tool for clustering and visualizing miRNA-mRNA interactions: *MMiRNA-Viewer*^*2*^ (http://bioinf1.indstate.edu/mmirna-viewer2/). *MMiRNA-Viewer*^*2*^ is a major update release compared to the *MMiRNA-Viewer* in the following aspect. First, compared to JavaScript-based *MMiRNA-Viewer*, *MMiRNA-Viewer*^*2*^ is supported by the Node JS Application (D3.js and JQuery: https://d3js.org) with browser side application prototype. The development of new technology brings graphic rendering speed boost, more dynamic manipulation of the miRNA-mRNA regulatory network, and diverse and flexible annotation and filter elements. Second, compared to *MMiRNA-Viewer* that manually incorporates the annotation information, *MMiRNA-Viewer*^*2*^ incorporates automatically all the annotation information to the nodes in the graph such as gene ontology, signal cascade pathways, cancer associated databases, which are specific to miRNA-gene functional interaction, their positive or negative effects on signaling pathways, and the experimentally validated cancer associated mutations.

## Materials and methods

### Generation of mRNA-miRNA input cluster pairs

We took the significant mRNA-miRNA target pairs obtained from RNA-Seq and miRNA-Seq data for 15 cancer types from The Cancer Genome Atlas (TCGA) (http://cancergenome.nih.gov) project. We used a similar approach adopted in our previous study [28, 29] to select input pairs. Specifically, we employed a computer C program to calculate the Pearson Correlation Coefficient (CC). The targets prediction outcomes were testified using Targetprofiler [30], TargetScan [31] and miRanda [32]. We wrote an R Script to compute the statistical significance (*P*-values and *Q*-values or False discovery rates (FDR)) for each calculated CC. We then ran a modified Louvain algorithm [27] employed by NetworkX (https://networkx.github.io/) to generate cluster pairs (unpublished data) used for input for *MMiRNA-Viewer*^*2*^.

### Comparison with other tools

We compared our *MMiRNA-Viewer*^*2*^ with other tools, miRMAP [26] and MAGIA2 [24], using the BRCA dataset processed from TCGA. The miRMAP tool doesn’t require mature miRNA ID as strict as MAGIA2. miRMAP only requires precursor miRNA, so we directly ran the miRMAP tool on the matched precursor miRNA and mRNA expression matrices in tumor generated using customized scripts. The two matrices were loaded into the miRMAP Java GUI to predict and visualize the miRNA–mRNA functional modules.

MAGIA2 [24] requires mature miRNA ID in order to take mRNA and miRNA profiles generated using platform-specific microarray probes, and thereby Perl scripts provided by [33] were used to process the miRNA isoform expression files to make the miRNA expression matrix with mature miRNA IDs. Using customized scripts, we generated and filtered the mRNA expression matrix to ensure that its samples were matched to samples in the mature miRNA expression matrix. Then the two matched expression matrices were uploaded to the MAGIA2 website.

## Results

### *MMiRNA-Viewer*^*2*^ web tool implementation

**Fig. 1:**
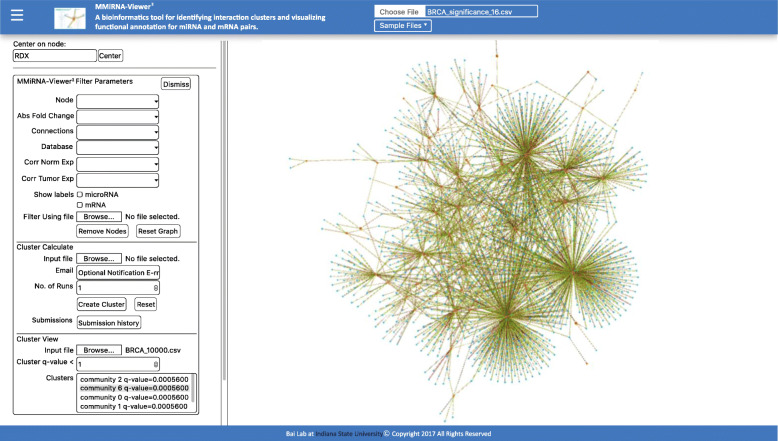
The visualization screenshot of *MMiRNA-Viewer*^*2*^

In Fig. 1, *MMiRNA-Viewer*^*2*^ architecture comprises four main sections: (i) upload data, (ii) filter parameters, (iii) calculate cluster, (iv) visualize cluster.

### Uploading data

The user selects the organism and uploads miRNA and gene (or mRNA) pair association data. The user will be taken to a file explorer view where the user can select a single file for upload. The specific format for the input data set is required. The file should be a text file with tab-delimited matrices (miRNA and mRNA pairs are rows and their various correlated values are columns). The raw expression files of miRNAs and genes for each individual sample were combined into huge sheets with miRNA and gene IDs as the first column. The correlation and target prediction information for significant miRNA-mRNA pairs between tumor and normal samples have been calculated and pre-selected. Besides *p*-values for Pearson coefficient, a false discovery rate (FDR, following Benjamini and Hochberg estimation method) was also calculated for each target pair. During the calculation, a filtering procedure of various parameters has been applied and series of quality checks are performed. To increase the confidence of prediction, we used three well-known target prediction algorithms to perform target prediction. At least one “Yes” prediction for a pair is required to be claimed to have a target relationship. The users could also use our available script pipeline (available on the website) to generate their input files if they provide two expression matrices (in the tab-delimited format) with genes and miRNAs on the rows and samples on the columns. For the convenience of comparison between tumor and normal cases, the pipeline also provided a step of selecting inversely correlated and oppositely expressed mRNA-miRNA pairs. This step can be skipped if the experimental design chosen by the user is not involved in the comparison.

Current *MMiRNA-Viewer*^*2*^ considers only one experimental design: Gene/transcript and miRNA expression data from the same biological samples with tumor and normal matched cases. No registration is required to use *MMiRNA-Viewer*^*2*^*.* Moreover, the design of *MMiRNA-Viewer*^*2*^ allows several users to perform analyses at any given location at the same time.

### Filtering parameter

After the input data are uploaded, user(s) can navigate through the network following miRNA and gene interactions to perform various analyses by specifying customized parameters in a series of steps. The users can search for a specific node (miRNA) and edge (mRNA) pairs in the graph by inserting the miRNA or gene name in the search box. After inputting an mRNA or miRNA name, the user can click the search button to center its position in the graph. The ID search is case-sensitive. Additionally, various filters can be applied to filter data at will. For example, when users select Node Filter as “mRNA” and Connections Filter as “> 10”, then only the nodes representing mRNA with the number of connected nodes greater than 10 will be highlighted in the graph.

### Calculating cluster

The input file consists miRNA and mRNA pair data in text format with the following information (i) expression correlations between mRNA and miRNA, (ii) mRNA and miRNA normal/tumor *p*-value and FDR values, and (iii) Fold Changes values for miRNA and mRNA pairs. *MMiRNA-Viewer*^*2*^ allows the user to download the output file that can be used for further processing (e.g. visualization), highlighting clusters according to the q-value cutoff selection. With this file, the user can just visualize their own interesting clusters and speculate on their functional relations.

### Visualizing cluster

The user-defined file/output file from the clustering result can be visualized by using the visualization option of *MMiRNA-Viewer*^*2*^. The processed output file which shows the miRNA and mRNA pairs. Users can click on two nodes that are connected with each other to get the annotation values in normal and tumor samples. Users can collapse and expand the legend on the top right corner in the graph by clicking the legend icon. The visualization graph presented by *MMiRNA-Viewer*^*2*^ is supported by the Node JS Application (D3.js and JQuery: https://d3js.org) with browser side application prototype. The algorithm for drawing out the graph starts with the links between mRNA and miRNA pairs. Links indicate the databases that validated the connection and normal/tumor correlation. The squares represent mRNAs while circles represent miRNAs.

### Database integration

Identifying disease-related miRNAs plays an important role in biomedical research. Computational models can identify novel miRNA–disease association in an efficiently and cheap way compared to experiment-based methods. There are also databases that have various data related to miRNAs for researchers to explore [34]. For miRNA and mRNA, we searched more than thirty databases to integrate into our developed tool and finally listed eight databases with comprehensive details for both miRNA and mRNA. For miRNA (i) The Human MicroRNA Disease Database (HMDD) [35] (ii) Somatic mutations altering microRNA-ceRNA interactions (SomamiR) [36] (iii) miRbase Database of published miRNA sequences and annotation (miRbase) [37] (iv) Kyoto Encyclopedia of Genes and Genomes (KEGG) [38] whereas for mRNA, (i) Database for Annotation, Visualization and Integrated Discovery (DAVID) [8] (ii) Kyoto Encyclopedia of Genes and Genomes (KEGG) [38] (iii) Catalogue of Somatic Mutations in Cancer (COSMIC) [39] (iiii) Cancertope [40] . The selection criteria were based on the (i) useful information required for microRNA and mRNA (ii) the availability of the database for academic research and (iii) diseases association for both miRNA and mRNA. Each gene is linked to HMDD [35], SomamiR [35, 36], miRbase [37], KEGG [38]. Each miRNA is linked to DAVID [15], KEGG [38] COSMIC [39] and Cancertope [40]. Furthermore, to allow efficient and systematic retrieval of statements from pathway databases, directly links results to KEGG.

**The Human MicroRNA Disease Database (HMDD)** [35] (http://cmbi.bjmu.edu.cn/hmdd) contains curated experiment-supported evidence for human miRNA (miRNA) and disease associations. In *MMiRNA-Viewer*^*2*^, we selected three module (i) HMDD Genetics, (ii) HMDD epigenetics, and (iii) HMDD target. All the three options provide information about miRNA, the disease name associated with respective miRNA, PMID and description of disease.

**Somatic mutation in MicroRNA (SomamiR)** [36] database gives details of cancer somatic mutations in miRNAs and their target sites that potentially alter the interactions between miRNAs and competing endogenous RNAs (ceRNA) including mRNAs, circular RNAs (circRNA) and lncRNA. From SomamiR, our selected module is “cancer associated genes that contain miRNA related somatic mutations.” Here, we extracted the data based on miRNA, DNA strand, Mutation ID, COSMIC mutation and description in a tabular form.

**The miRbase** (http://www.mirbase.org/) database [37] published miRNA sequences and annotation. Each entry in the miRBase Sequence database represents a predicted hairpin portion of a miRNA transcript (termed mir in the database), with information on the location and sequence of the mature miRNA sequence (termed miR). Both hairpin and mature sequences are available for searching and browsing, and entries can also be retrieved by name, keyword, references, and annotation. The data extracted from miRbase consist of miRNA, Accession number, and sequences. The Accession number provides a hyperlink that is connected to the miRbase server for further details.

**Kyoto Encyclopedia of Genes and Genomes (KEGG)** [38] is a database resource for understanding high-level functions and utilities of the biological system, such as the cell, the organism and the ecosystem, from molecular-level information, especially large-scale molecular datasets generated by genome sequencing and other high-throughput experimental technologies. For miRNA, we integrated KEGG cancer pathways for both miRNA and mRNA categories.

**The Database for Annotation, Visualization and Integrated Discovery (DAVID)** v6.8 [15] comprises a full Knowledgebase update to the sixth version of our original web-accessible programs. DAVID now provides a comprehensive set of functional annotation tools for investigators to understand biological meaning behind large list of genes. The DAVID information is based on mRNA, Gene Name, Chromosome location, BIND, Prosite, Panther and Reactome pathway in a tabular form.

**The Catalogue of Somatic Mutations in Cancer (COSMIC)** [39] is the world’s largest and most comprehensive resource for exploring the impact of somatic mutations in human cancer. We integrated three modules from COSMIC namely (i) Cancer Gene Census, (ii) Resistance Mutation, and (iii) Target Mutant Exports. The information from above-listed modules mainly consists of gene name, genome location, the primary site of cancer, Chromosome band, somatic mutations, Transcript ID and gene length.

**Cancertope** [40] is a Platform for Designing Genome-Based Personalized Immunotherapy or Vaccine against Cancer. The integrated modules from Cancertope are (i) Epitope Search, (ii) Data Retrieval, and (iii) Browse Gene. The main reason of integrated Cancertope is to provide the immunological connection of diseases with miRNA and mRNA which may help the user to shortlist the candidate genes/miRNA for designing vaccination against cancer.

### Comparison among *MMiRNA-Viewer*^*2*^, miRMAP, and MAGIA2

#### *MMiRNA-Viewer*^*2*^ result

From the BRCA data set, the *MMiRNA-Viewer*^*2*^ detected eight significant clusters that has q values less than 0.05. The cluster ID, q value, miRNA-mRNA pair number, mRNA number, and miRNA number are summarized in Table 1.

**Table 1:** Clusters detected by the *MMiRNA-Viewer*^*2*^. Each cluster are composed of miRNA and miRNA nodes and their interactions

Cluster ID	Q value	miRNA-mRNA pair number	mRNA number	miRNA number
1	1.00E-05	734	572	21
3	1.00E-05	538	491	9
6	1.00E-05	1308	698	77
7	1.00E-05	740	513	14
8	1.00E-05	471	345	17
9	1.00E-05	1019	570	58
11	1.00E-05	636	489	22
14	0.000189998	412	342	24

**Fig. 2:**
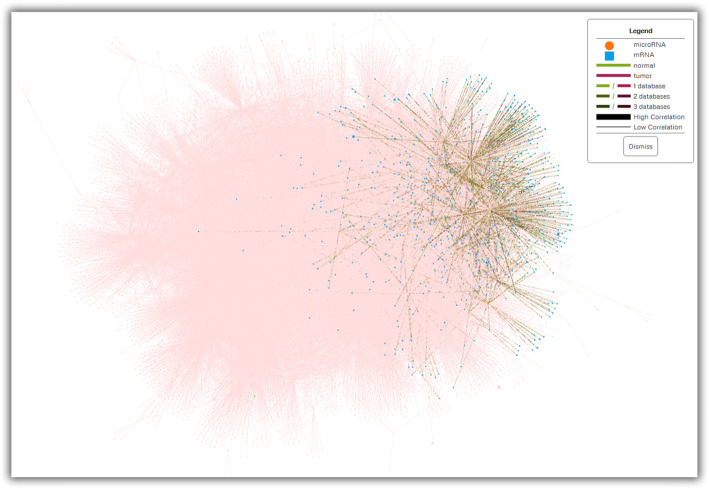
cluster/community 1 shown as an example. miRNAs, mRNAs, and interactions that are not included in the cluster 1 are faded as pink color in the background

The *MMiRNA-Viewer*^*2*^ is an interactive visualization tool so the clusters can be shown interactively online. When a specific cluster is selected, all other miRNA, mRNA, and connection will be faded to become the background so that the selected cluster is outstanding. Cluster 1 is shown as an example in Fig. 2.

#### miRMAP result

**Fig. 3:**
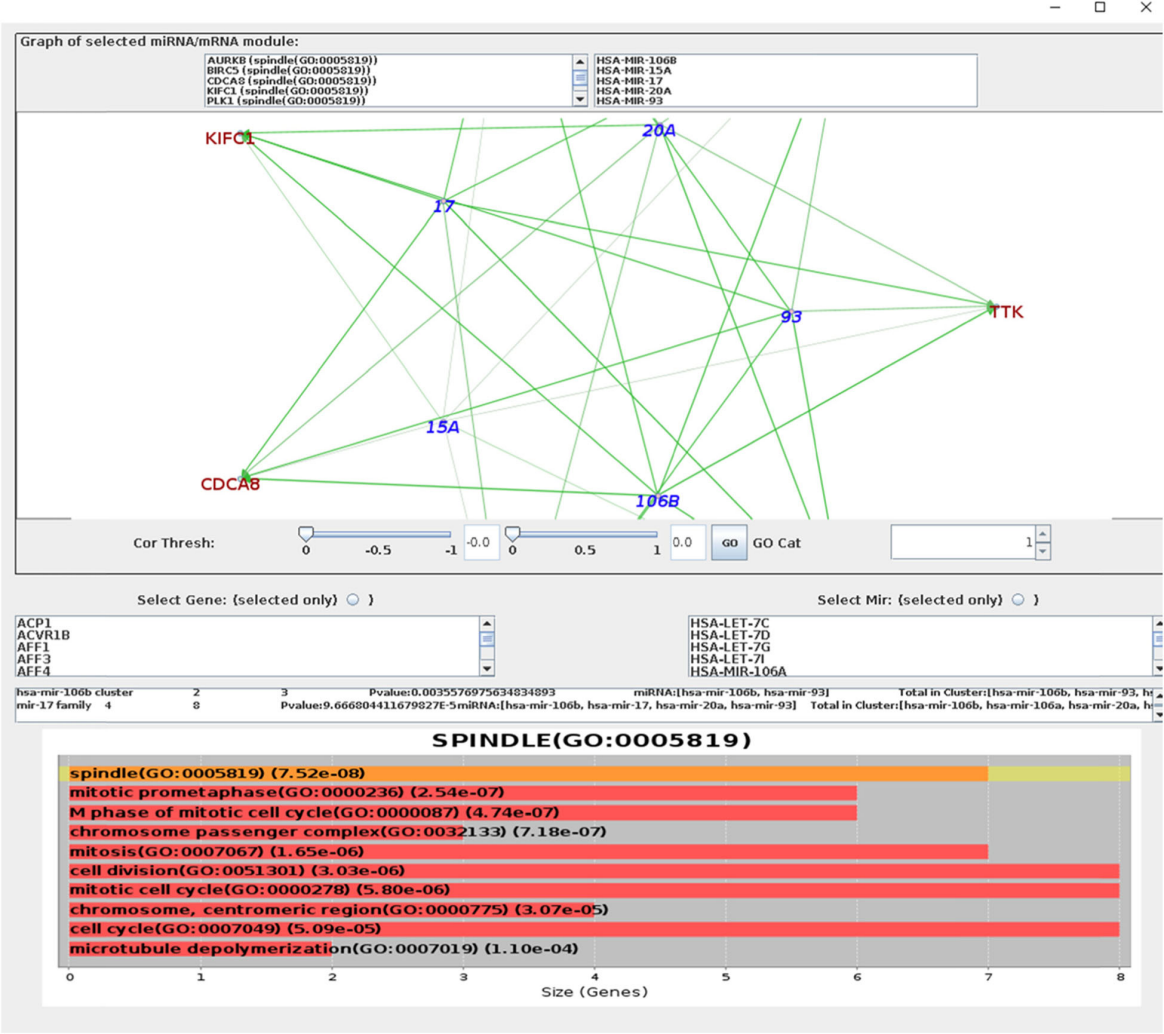
a cluster detected by miRMAP. The enriched GO terms are also listed

All the parameters followed the miRMAP default settings. Finally, 25 miRNA-mRNA functional modules (i.e. miRNA-mRNA biclusters) were produced, in which there were GO term cell proliferation and cell cycle, suggesting some genes could be correlated to breast cancer. One cluster detected by miRMAP is visualized in Fig. 3.

#### MAGIA2 result

**Fig. 4:**
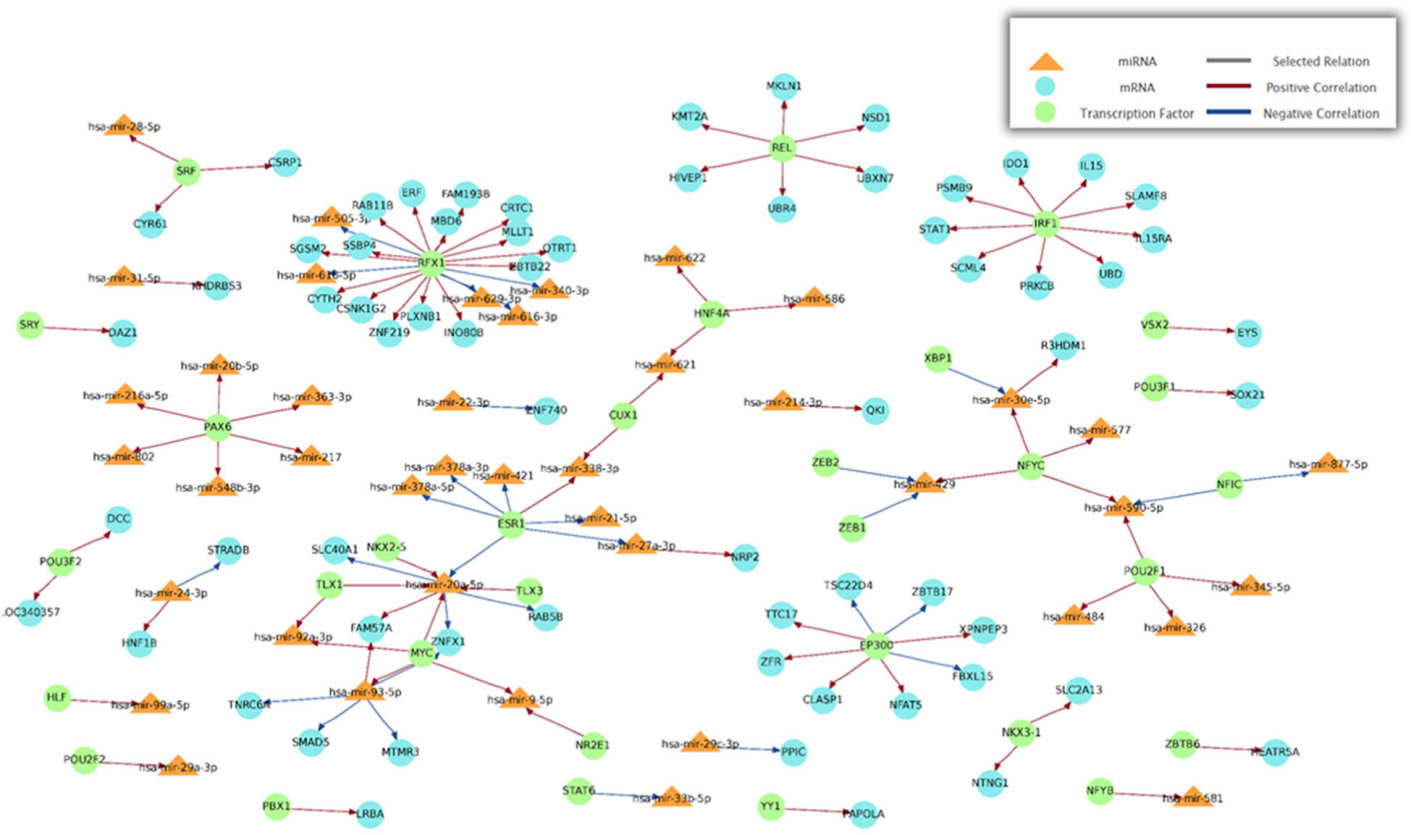
miRNA-mRNA regulatory network detected by MAGIA2 using Pearson correlation as their interaction measurement

All the predictor parameters followed the MAGIA2 default settings except for excluding the microcosm and rna22, which had ID conversion issues. The Pearson correlation method was adopted. The regulatory network of miRNAs, mRNAs, and transcription factors constructed by MAGIA2 is shown in Fig. 4.

#### Differences among *MMiRNA-Viewer*^*2*^, miRMAP, and MAGIA2 results

*MMiRNA-Viewer*^*2*^ can visualize the clusters interactively. Users can see how the detected clusters stand out of the background miRNA-mRNA pairs. Users can directly see the functional annotation of miRNAs and mRNAs that are related to diseases by right click on the nodes. Users can zoom in and zoom out or drag the miRNA-mRNA network. miRMAP, by default can detect 25 miRNA-mRNA modules. The bicluster setting of miRMAP can highlight the specific pattern of miRNA-mRNA interactions, but not all miRNAs and mRNAs interact in the bicluster pattern. The MAGIA2 can detect miRNA-mRNA pairs, distinguish mRNAs that are targeted by miRNAs and mRNAs that are transcription factors that regulate miRNAs. However, the MAGIA2 results can not cluster the miRNA and mRNA pairs based on their expression correlation coefficient.

## Discussions

*MMiRNA-Viewer*^*2*^ is a software that can calculate the significant pairs of mRNA-miRNA in cases compared to controls. By comparison, MAGIA2 and miRMAP can only consider one condition. *MMiRNA-Viewer*^*2*^ can highlight detected clusters from clustering algorithm results. Users can also upload their custom clustering algorithm results to the web server.

*MMiRNA-Viewer*^*2*^ outperforms MAGIA2 and miRMAP in computational visualization because *MMiRNA-Viewer*^*2*^ adopted advanced graphics technology Node JS Application (D3.js and JQuery) with browser side application prototype. So *MMiRNA-Viewer*^*2*^ can efficiently, interactively, and dynamically render these miRNA-mRNA interaction network. For example, the targeting intensity, miRNA-mRNA correlation coefficient in tumor as well as in normal, functional annotation of miRNA and mRNA nodes, and so on can all be shown in the browser. In *MMiRNA-Viewer*^*2*^ web tool, the user can also filter the interactions based on correlation of tumor and normal data (experimental vs. control data). The user can also select/deselect the cluster for visualization based on its *P*-value to compare different significant clusters with cutoff value simultaneously in *MMiRNA-Viewer*^*2*^ visualization tool. The additional data listed with visualization like Tumor FDR, Normal FDR, Tumor Correlation Coefficient, and Normal Correlation Coefficient are other additional parameters for the user to check the expression level of the selected cluster. By comparison, the miRMAP and MAGIA2 don’t bear such enriched visualization elements.

The miRMAP and MAGIA2 focus on the functional analysis such as DAVID or GO terms, etc. Similarly but more conveniently, the *MMiRNA-Viewer*^*2*^ establish a direct connection with interacting databases and ontologies such as DAVID [15], HMDD [35], SomamiR [36], miRbase [37], KEGG [38] and COSMIC [39] and Cancertope [40] to extract the functional annotation. Thereby, *MMiRNA-Viewer*^*2*^ serves as a multitasking platform, on which users can identify significant interaction clusters and retrieve functional and cancer-associated information for miRNA-mRNA pairs between tumor and normal samples. *MMiRNA-Viewer*^*2*^ gives a detailed presentation of miRNA associated with several types of human cancers, and provides a comprehensive analysis of gene ontology and miRNA-cancer associations. *MMiRNA-Viewer*^*2*^ helps the user to convert the abundance of biological data into meaningful information regarding miRNA roles in cancer association and to get directly specific biological function information instead of retrieving information from multiple databases manually and respectively.

## Conclusions

Utilizing the next generation sequencing data of miRNA and mRNA expression profiles in tumor and in normal, our tool *MMiRNA-Viewer*^*2*^ provided a list of candidate cancer-associated genes and miRNAs with their biological functions, and thereby could benefit the current research and/or clinical practice. Our proposed method is applicable across a range of diseases and cancers and has advantages over existing tools. This will likewise contribute to new bioinformatics methodologies for identifying cancer driver genes in personal genomes, in which clinicians seek to develop better treatment strategies. In the future, we plan to explore more algorithms to cluster interactions in both tumor and normal samples of various cancer types.

## Data Availability

*MMiRNA-Viewer*^*2*^ can be accessed at http://bioinf1.indstate.edu/mmirna-viewer2/ and is freely available to use. The software and all data supporting the software is available at https://drive.google.com/file/d/15g78TNmFaSETmMs8n3xS4O3RrqLjf9l1/view?usp=sharing.

## Abbreviations

BRCA: Breast cancer
TCGA: The Cancer Genome Atlas
DAVID: The Database for Annotation, Visualization and Integrated Discovery (DAVID)
HMDD: The Human microRNA Disease Database
SomamiR: Somatic mutation altering microRNA-ceRNA interactions
KEGG: Kyoto Encyclopedia of Genes and Genomes
COSMIC: Catalogue of Somatic Mutation in Cancer
